# Suit for Malpractice

**Published:** 1870-03

**Authors:** 


### Suit for Malpractice.

We have recently had an opportunity to defend our practice in a case of oblique fracture of the bones of the leg, in the person of a pauper we attended, because a neighbor represented that five doctors had visited her and left without dressing, urging us "for God sake to go and set the poor woman's leg." It was shown on the trial that our dressings were removed by a neighbor, night and morning, that several *quacks* besides ourselves were also in attendance during nearly the entire period of treatment, that the leg was perfect in every other respect except $\frac{3}{4}$ to $\frac{7}{8}$ inch shortening, this being as little as could be expected in any case of so great obliquity; that the care was ample and satisfactory in every respect. It was also shown that the leg was as capable of service as ever, though patient still used crutch and cane, when out on the street, but could carry water, even up stairs without difficulty when at home. She had a vegetable doctor and a cancer doctor who testified to shortening and dressing and various other items, showing to the most unobserving that they knew nothing at all about it. Her only point was a crutch and cane, which had been carried a whole year in order to have them on hand as evidence.

There was nothing in the case of any professional interest, only the simple illustration that physicians are liable to the expense of defense, in cases where there is not the slightest ground for complaint. This condition has long been recognized, and has been believed to grow mainly out of want of fidelity to each other, on the part of the members of the medical profession. We have formerly, in Buffalo, suffered from this cause alone, personal quarrels and jealousies in the profession being the main spring of action and the corner stone of strength.

We are happy to announce, for the honor of the profession in Buffalo, that no "roots of bitterness" were shown, that the testimony of the regular profession on both sides of the case, was harmonious, and to the entire justification of the practice adopted, and the perfection of the results obtained. To be able to speak thus is a pleasure in which all right minded physicians will participate.

:o:

We have lately received from Dr. Jerome Kidder, one of his electrical instruments, designed for physicians who desire to make use of electricity as a therapeutical agent. In Europe, and especially in Germany, great attention is being given in the direction of electro-therapeusis, and it is claimed with great earnestness, that satisfactory results have been attained. While our personal efforts in this direction, heretofore, have not been remarkable for success, still we are not ready to denounce more accurate experimenters and their carefully stated observations. Dr. Kidder's machine is recommended by the authors as being the one which affords both the direct and the induced currents with their modifications.

